# Progression of Aortic Arch Calcification Over 1 Year Is an Independent Predictor of Mortality in Incident Peritoneal Dialysis Patients

**DOI:** 10.1371/journal.pone.0048793

**Published:** 2012-11-07

**Authors:** Mi Jung Lee, Dong Ho Shin, Seung Jun Kim, Hyung Jung Oh, Dong Eun Yoo, Kwang Il Ko, Hyang Mo Koo, Chan Ho Kim, Fa Mee Doh, Jung Tak Park, Seung Hyeok Han, Tae-Hyun Yoo, Kyu Hun Choi, Shin-Wook Kang

**Affiliations:** 1 Department of Internal Medicine, College of Medicine, Yonsei University, Seoul, Korea; 2 Severance Biomedical Science Institute, Brain Korea 21, Yonsei University, Seoul, Korea; Maastricht University Medical Center, The Netherlands

## Abstract

**Backgrounds and Aims:**

The presence and progression of vascular calcification have been demonstrated as important risk factors for mortality in dialysis patients. However, since the majority of subjects included in most previous studies were hemodialysis patients, limited information was available in peritoneal dialysis (PD) patients. Therefore, the aim of this study was to investigate the prevalence of aortic arch calcification (AoAC) and prognostic value of AoAC progression in PD patients.

**Methods:**

We prospectively determined AoAC by chest X-ray at PD start and after 12 months, and evaluated the impact of AoAC progression on mortality in 415 incident PD patients.

**Results:**

Of 415 patients, 169 patients (40.7%) had AoAC at baseline with a mean of 18.1±11.2%. The presence of baseline AoAC was an independent predictor of all-cause [Hazard ratio (HR): 2.181, 95% confidence interval (CI): 1.336–3.561, *P* = 0.002] and cardiovascular mortality (HR: 3.582, 95% CI: 1.577–8.132, *P* = 0.002). Among 363 patients with follow-up chest X-rays at 12 months after PD start, the proportion of patients with AoAC progression was significantly higher in patients with baseline AoAC (64.2 vs. 5.3%, *P*<0.001). Moreover, all-cause and cardiovascular death rates were significantly higher in the progression groups than in the non-progression group (*P*<0.001). Multivariate Cox analysis revealed that AoAC progression was an independent predictor for all-cause (HR: 2.625, 95% CI: 1.150–5.991, *P* = 0.022) and cardiovascular mortality (HR: 4.008, 95% CI: 1.079–14.890, *P* = 0.038) in patients with AoAC at baseline.

**Conclusions:**

The presence and progression of AoAC assessed by chest X-ray were independently associated with unfavorable outcomes in incident PD patients. Regular follow-up by chest X-ray could be a simple and useful method to stratify mortality risk in these patients.

## Introduction

Cardiovascular disease is the most common cause of morbidity and mortality in patients with end-stage renal disease (ESRD) [1]. Since traditional risk factors, such as advanced age, hypertension, diabetes, smoking, and dyslipidemia, cannot fully account for the high prevalence of cardiovascular disease, uremia-related factors, including inflammation and oxidative stress, have been implicated in the pathogenesis of cardiovascular disease in ESRD patients [2]. Recently, accumulating evidence has shown that disturbances in calcium-phosphorus metabolism also play a pivotal role in cardiovascular disease, partly via the development of vascular calcification [2], [3], [4].

Vascular calcification is not uncommon in general elderly population; 20–30% of people older than 65 years have calcification in the aorta [5]. In patients with chronic kidney disease (CKD), this proportion is reported to be substantially higher; more than one half of CKD patients even before the start of dialysis and up to 80–90% of ESRD patients have some form of vascular calcification [6], [7]. Previous studies have revealed vascular calcification is independently associated with all-cause and cardiovascular mortality in both general population and ESRD [3], [8], [9], [10], [11]. Moreover, since vascular calcification progresses rapidly in dialysis patients, ESRD patients with the progression of vascular calcification are demonstrated to have an unfavorable outcome [12]. Therefore, not only the identification of vascular calcification but also risk stratification of patients by the changes in vascular calcification may be important for clinicians to manage dialysis patients.

To date, a number of techniques are available to detect vascular calcification. Electron beam computed tomography (EBCT), multi-slice CT (MSCT), planar X-ray (such as plain X-ray of lateral abdomen, pelvis, and hands), 2D ultrasonography, and echocardiography have been used to assess vascular calcification [6], [9], [10], [13], [14], [15], [16], [17]. Among these, EBCT and MSCT are well-validated noninvasive imaging methods that are considered the golden standard for quantifying vascular calcification. However, EBCT and MSCT cannot be routinely performed due to the relatively high cost of testing and exposure to a high radiation dose [16]. Recently, aortic arch calcification (AoAC) in plain chest X-rays was found to reflect the magnitude of whole aortic calcification in general population and dialysis patients [15], [16]. In addition, several previous studies showed that AoAC was an independent predictor of cardiovascular events and that AoAC progression was significantly associated with increased cardiovascular mortality in patients with ESRD [3], [11], [18], [19]. However, since the majority of subjects included in most previous studies were ESRD patients on hemodialysis (HD), little is known about the prevalence, natural history, and prognostic value of vascular calcification in peritoneal dialysis (PD) patients. In the present study, we investigated the prevalence of AoAC at PD initiation and the frequencies of AoAC progression or regression during the first year after PD. The impact of AoAC progression on all-cause and cardiovascular mortality was also determined.

## Methods

### Ethics Statement

The study was carried out in accordance with the Declaration of Helsinki and approved by the Institutional Review Board of Yonsei University Health System Clinical Trial Center. We obtained informed written consent from all participants involved in our study.

### Patients

All consecutive ESRD patients over 18 years of age who started PD at Yonsei University Health System between January 2005 and June 2010 were initially included in this prospective observational study. Among a total of 530 incident PD patients, patients with PD duration of less than 3 months, active infection, malignancy, and decompensated liver cirrhosis were excluded. Thus, the remaining 415 patients were included in the final analysis.

### Demographic and Clinical Data Collection

A well-trained examiner used a questionnaire at the time of PD start to collect demographic data. Traditional cardiovascular risk factors such as age, hypertension, diabetes mellitus, smoking history, and previous history of cardiovascular disease were recorded. In smokers, the amount of smoking was expressed as pack-years; the product of the number of cigarette packs consumed per day by the duration of smoking (years). Cardiovascular disease was defined as a history of coronary, cerebrovascular, or peripheral vascular disease: coronary disease was defined as a history of angioplasty, coronary artery bypass grafts, myocardial infarction, or angina and cerebrovascular disease as a history of transient ischemic attack, stroke, or carotid endarterectomy, while peripheral vascular disease was defined as a history of claudication, ischemic limb loss and/or ulceration, or peripheral revascularization procedure. Patients were weighed in light clothing and height was measured with no shoes. Body mass index (BMI) was calculated as weight/height^2^ (kg/m^2^). Blood was drawn after a 12-hour overnight fasting, and the following laboratory data were measured from blood samples: hemoglobin, blood urea nitrogen, creatinine, calcium, phosphorus, albumin, total cholesterol, triglyceride, low density lipoprotein (LDL)-cholesterol, high density lipoprotein (HDL)-cholesterol, and intact parathyroid hormone (iPTH) concentrations. In addition, high sensitivity C-reactive protein (hs-CRP) levels were determined by a latex-enhanced immunoephelometric method using a BN II analyzer (Dade Behring, Newark, DE, USA). To reflect the actual situation, usual overnight dialysate volume and glucose concentrations were not changed for this study. Kt/V urea was determined from the total loss of urea nitrogen in spent dialysate using PD Adequest 2.0 for Windows software (Baxter Healthcare, Deerfield, Illinois, USA). The modified peritoneal equilibration test was performed with 4.25% glucose dialysis solution as described previously [20] and the dialysate-to-plasma creatinine (D/P Cr) and glucose (D/D0 glucose) concentration ratios at 4 hours of dwell were used to describe the peritoneal transport characteristics; high, high average, low average, and low.

### Assessment of AoAC by Chest X-ray

To determine AoAC extent, two trained medical doctors blinded to the patients’ clinical data reviewed posterior-anterior plain chest X-rays taken at the start of PD using a specific scale developed by Ogawa et al [16]. This scale, which divides the aortic arch into 16 sections by circumference, was attached to the aortic arch on chest X-rays and the number of sectors was divided by 16. AoAC scores (AoACS) were calculated after multiplication by 100 to express results as a percentage. To confirm the intra-reader variability, randomly selected 100 chest X-rays were reexamined by the same reader. The median intra-class correlation coefficient for AoACS was 0.91 [95% confidence interval (CI): 0.71 to 0.99] and 0.90 (95% CI: 0.69 to 0.98) in two readers. In addition, any discrepancies between the two observers were resolved by an independent third reader. Progression of AoAC was defined as an increase in AoACS on the follow-up chest X-ray taken 1 year after PD initiation.

### Follow-up and Endpoints

All patients included in this study were regularly followed-up at the PD clinic, and all deaths and hospitalization were recorded in the serious adverse events database. Mortality events were retrieved from the database and carefully reviewed to determine all-cause and cardiovascular mortality. Cardiovascular mortality was considered death from myocardial infarction or ischemia, congestive heart failure, pulmonary edema, and cerebral hemorrhage or vascular disorder.

Among 415 patients, follow-up chest X-rays at 12 months were not available in 52 patients; 30 died within 12 months of PD start, 11 changed dialysis modality to HD, 9 underwent kidney transplantation, and 2 were transferred to other PD units. Therefore, the association between the progression of AoAC and survival was analyzed in 363 patients.

### Statistical Analysis

Statistical analysis was performed using SPSS for Windows version 18.0 (SPSS Inc., Chicago, IL, USA). Continuous variables were expressed as mean ± SD, and categorical variables were expressed as a number (percentage). Since hs-CRP did not yield a Gaussian distribution, log values were used. In the first analysis, 415 patients were divided into two groups according to the presence of AoAC at baseline. To determine differences between the two groups, a Student’s t-test and the chi-square test were performed for continuous variables and categorical variables, respectively. Multivariate binary logistic regression models were used to identify significant determinants of AoAC presence at PD initiation. Cumulative survival curves were generated by the Kaplan-Meier method, and between-group survival was compared by a log-rank test. Independent prognostic values of AoAC at baseline for all-cause and cardiovascular mortality were ascertained by Cox proportional hazards models, which included only the significant variables in univariate analysis. Meanwhile, the progression of AoAC was focused in the second analysis. In the second analysis, mean values of the biochemical parameters during the first year of PD were used. Pearson’s correlation analysis was performed to estimate association between the changes in AoACS and other continuous variables. Multivariate binary logistic regression models, which included significant variables in univariate analysis, were constructed to determine significant independent predictors of AoAC progression. Subgroup analysis was also performed according to the presence of baseline AoAC. The impact of AoAC progression on patient outcome was examined by the Kaplan-Meier method and Cox proportional hazards regression analysis. Significant variables in univariate analysis, traditional risk factors (age, sex, and diabetes mellitus), and factors associated with inflammation and nutrition (serum hs-CRP and albumin concentrations) were included in multivariate Cox proportional hazard models. A *P* value less than 0.05 was considered statistically significant.

## Results

### Clinical Characteristics According to the Presence of AoAC at Baseline

Baseline patient characteristics according to the presence of AoAC at baseline are shown in Table 1. The mean age was 55.8±13.8 years (21–80 years), and 234 patients (56.3%) were male. Of 415 patients, 169 patients (40.7%) had AoAC at baseline with a mean AoACS of 18.1±11.2%. Diabetic nephropathy was the most common cause of ESRD, followed by chronic glomerulonephritis in both groups. The mean age, the proportion of patients with diabetes and previous history of cardiovascular disease, and the proportion of patients taking lipid-lowering agents and β-blockers were significantly higher in patients with AoAC at baseline. In addition, compared to patients without baseline AoAC, total cholesterol, iPTH, and albumin concentrations were significantly lower, while hs-CRP levels were significantly higher in the baseline AoAC present group. Moreover, even though the proportion of smoker was significantly lower, the mean amount of smoking was significantly greater in patients with baseline AoAC. Among 224 patients (53.9%), who performed echocardiography at baseline, the ejection fraction was significantly lower in patients with baseline AoAC compared to the baseline AoAC absent group. On the other hand, there were no significant differences in peritoneal membrane transport characteristics, weekly Kt/V urea, systolic blood pressure, BMI, calcium-phosphate (Ca x P) product values, and the use of phosphate binders between the two groups.

**Table 1:** Baseline characteristics of the patients with and without aortic arch calcification (AoAC).

Characteristics	With AoAC	Without AoAC	*P*
Number (%)	169 (40.7%)	246 (59.3%)	
Age (years)	66.7±9.3	52.1±13.1	<0.001
Male, *n* (%)	88 (52.0%)	146 (59.3%)	NS
Diabetes mellitus, *n* (%)	104 (61.5%)	92 (37.3%)	<0.001
Primary renal disease, *n* (%)			NS
Glomerulonephritis	38 (22.4%)	73 (29.6%)	
Diabetes mellitus	86 (50.9%)	84 (34.1%)	
Hypertensive nephrosclerosis	12 (7.1%)	21 (8.5%)	
Polycystic kidney disease	1 (0.6%)	4 (1.6%)	
Others/Unknown	32 (18.9%)	64 (26.0%)	
Peritoneal equilibration test, *n* (%)			NS
High	7 (4.1%)	24 (9.8%)	
High average	123 (72.7%)	126 (51.2%)	
Low average	34 (20.1%)	90 (36.5%)	
Low	5 (2.9%)	6 (2.4%)	
Kt/V urea (per week)	2.3±0.5	2.5±0.7	NS
Cardiovascular disease, *n* (%)	94 (55.6%)	51 (20.7%)	<0.001
Ejection fraction (%)	52.8±17.5	61.4±9.8	0.03
History of smoking, *n* (%)	41 (24.2%)	87 (35.3%)	0.02
Amount of smoking (pack-years)	35.1±24.0	24.1±18.2	0.03
Systolic blood pressure (mmHg)	139.3±21.8	139.8±19.8	NS
BMI (kg/m^2^)	22.6±3.0	22.6±3.1	NS
Hemoglobin (g/dL)	9.2±1.4	9.2±1.6	NS
Total cholesterol (mg/dL)	147.7±43.5	158.8±43.4	0.02
Ca × P product (mg^2^/dL^2^)	41.6±12.7	43.9±12.6	NS
iPTH (pg/mL)	138.4±123.8	213.5±176.0	<0.001
Albumin (g/dL)	3.4±0.5	3.5±0.6	0.008
Log hs-CRP (mg/L)	0.1±0.6	–0.2±0.9	<0.001
Lipid-lowering therapy, *n* (%)	80 (47.3%)	65 (26.4%)	<0.001
Antihypertensive drugs, *n* (%)			
RAS blockers	128 (75.7%)	189 (76.8%)	NS
Beta-blockers	105 (62.1%)	111 (45.1%)	0.03
Calcium channel blockers	107 (63.3%)	150 (60.9%)	NS
Phosphate binders, *n* (%)			NS
Calcium-based	88 (52.0%)	126 (51.2%)	
Non calcium-based	13 (7.6%)	19 (7.7%)	

Data are expressed as mean ± standard deviation or number of patients (percent).

Kt/V, fractional urea clearance; BMI, body mass index; Ca, calcium; P, phosphate; iPTH, intact parathyroid hormone; hs-CRP, high sensitivity C-reative protein; RAS, Renin-angiotensin system; NS, not significant.

### Association of Various Parameters with the Presence of AoAC at Baseline

In univariate analysis, age, diabetes mellitus, previous history of cardiovascular disease, smoking, lipid-lowering therapy, serum albumin, iPTH, and hs-CRP concentrations were significantly associated with the presence of AoAC at baseline. Multivariate binary logistic regression analysis revealed that age [odds ratio (OR): 1.101, 95% CI: 1.066–1.138, *P*<0.001] and previous history of cardiovascular disease (OR: 2.084, 95% CI: 1.006–4.314, *P* = 0.048) were significant independent factors associated with the presence of AoAC at baseline.

### Presence of AoAC at Baseline as an Independent Risk Factor for All-cause and Cardiovascular Mortality

**Figure 1:**
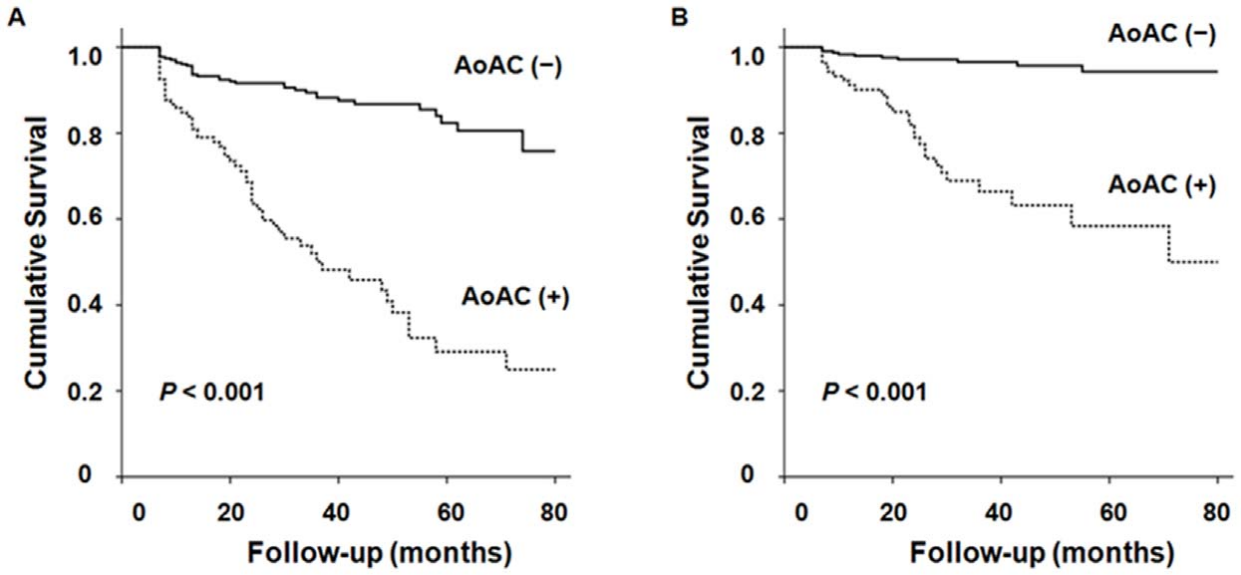
Kaplan-Meier analysis of (A) all-cause and (B) cardiovascular mortality in 415 patients. Patients with baseline aortic arch calcification (AoAC) showed significantly higher all-cause and cardiovascular mortality than those without (both log-rank test, *P*<0.001).

During a mean follow-up duration of 34.2±20.4 months, 90 patients (21.7%) died. Among them, 39 patients (43.3%) died from cardiovascular causes. Both the all-cause and cardiovascular mortality-free survival rates were significantly lower in patients with baseline AoAC (log-rank test, *P*<0.001) (Figure 1). Univariate Cox proportional hazard analysis showed older age, presence of diabetes and previous cardiovascular disease, usage of lipid-lowering medication, increased Ca × P products and hs-CRP levels, decreased albumin concetrations, and presence of AoAC at baseline were significant risk factors for all-cause and cardiovascular mortality. In multivariate Cox analysis, the presence of baseline AoAC was revealed as a significant independent predictor of all-cause [Hazard ratio (HR): 2.181, 95% CI: 1.336–3.561, *P* = 0.002] and cardiovascular mortality (HR: 3.582, 95% CI: 1.577–8.132, *P* = 0.002). Previous history of cardiovascular disease and higher hs-CRP levels were also found to be independent risk factors for all-cause and cardiovascular mortality. In contrast, older age was independently associated only with all-cause mortality (Table 2).

**Table 2:** Multivariate Cox’s proportional hazard models of baseline aortic arch calcification (AoAC) all-cause and cardiovascular mortality.

	All- cause mortality			Cardiovascular mortality		
	HR	95% CI	*P*	HR	95% CI	*P*
Age (years)	1.048	1.022–1.074	<0.001	1.028	0.988–1.069	NS
Male gender	1.136	0.660–1.954	NS	0.554	0.254–1.206	NS
Diabetes mellitus	1.071	0.679–1.690	NS	0.772	0.389–1.532	NS
Cardiovascular disease	2.000	1.143–3.500	0.015	3.807	1.441–10.054	0.007
History of smoking	0.928	0.520–1.657	NS	0.522	0.226–1.209	NS
Lipid-lowering therapy	1.027	0.629–1.676	NS	1.453	0.688–3.071	NS
Ca×P (mg^2^/dL^2^)	0.989	0.970–1.007	NS	1.002	0.972–1.032	NS
Albumin (g/dL)	0.763	0.520–1.118	NS	0.707	0.389–1.285	NS
Log hs-CRP (mg/L)	1.725	1.257–2.367	<0.001	1.769	1.044–2.996	0.034
Baseline AoAC	2.181	1.336–3.561	0.002	3.582	1.577–8.132	0.002

Ca, calcium; P, phosphate; hs-CRP, high sensitivity C-reative protein; HR, hazard ratio; CI, confidence interval; NS, not significant.

### Progression of AoAC: Subgroup Analysis According to the Presence of Baseline AoAC

Follow-up chest X-rays at 12 months after PD start were available in 363 patients. Among them, 140 patients (38.5%) had AoAC at baseline and 223 patients (61.5%) did not. The progression of AoAC was significantly more observed in patients with AoAC at baseline (*P*<0.001). Among 140 patients with AoAC at baseline, 90 patients (64.2%) experienced AoAC progression, whereas AoAC progressed in only 12 (5.3%) out of 223 patients without baseline AoAC. Two hundred eleven patients with AoACS of zero at baseline remained free of AoAC during the 12-month follow-up.

Pearson’s correlation analysis revealed that changes in AoACS were significantly associated with baseline AoACS (*r* = 0.389, *P*<0.001), age (*r* = 0.301, *P*<0.001), and time-averaged hs-CRP (*r* = 0.167, *P* = 0.001) and calcium concentrations (*r* = 0.124, *P* = 0.02). In multivariate binary logistic regression analysis, baseline AoACS (OR: 1.803, 95% CI: 1.383–2.349, *P*<0.001), age (OR: 1.058, 95% CI: 1.016–1.101, *P* = 0.006), and hs-CRP levels (OR: 1.904, 95% CI: 1.180–3.070, *P* = 0.008) were found to be independent risk factors associated with AoAC progression. Since the baseline AoACS was significantly correlated with AoAC progression, subgroup analysis was performed to clarify the independent predictor for AoAC progression in patients with and without baseline AoAC. In patients with AoAC at baseline, there was a significant correlation between hs-CRP concentrations and the changes in AoACS (*r* = 0.248, *P* = 0.02), while changes in AoACS were significantly associated with age (*r* = 0.124, *P* = 0.04) and hs-CRP levels (*r = *0.126, *P* = 0.036) in patents without baseline AoAC. However, the changes in Ca × P products and iPTH concentrations did not correlate with changes in AoACS in both subgroups.

Similar findings were observed in binary logistic regression analysis. In patients with AoAC at baseline, univariate analysis reavealed that diabetes mellitus, previous cardiovascular disease, lipid-lowering therapy, hs-CRP levels, and baseline AoACS were significantly associated with AoAC progression. In multivariate binary logistic regression models, baseline AoACS (OR: 1.234, 95% CI: 1.104–5.197, *P* = 0.027) and hs-CRP levels (OR: 2.238, 95% CI: 1.051–4.767, *P* = 0.037) were independent predictors of AoAC progression after adjustment for confounders. On the other hand, in patients without baseline AoAC, age, previous cardiovascular disease, the use of lipid-lowering drugs, and hs-CRP levels were significant predictors of AoAC progression in univariate analysis. Multivariate binary logistic regression models demonstrated that age (OR: 1.063, 95% CI: 1.014–1.113, *P* = 0.002) and hs-CRP concentrations (OR: 1.294, 95% CI: 1.019–4.581, *P* = 0.035) were significant risk factors for AoAC progression. However, peritoneal membrane transport characteristics, weekly Kt/V urea, Ca x P products, iPTH concentrations, and the use of phosphate binders were not significantly associated with AoAC progression in both subgroups.

### Progression of AoAC as an Independent Risk Factor for Mortality

In patients with AoAC at baseline, all-cause and cardiovascular death rates were significantly higher in the AoAC progression group (19.8 vs. 8.6 and 11.0 vs. 3.8 per 100 Person-Years, respectively, *P*<0.001). Results were similar even when the analysis was performed using only patients without baseline AoAC. In the progression groups, all-cause and cardiovascular death rates were 11.1 and 4.4 per 100 Person-Years, respectively. These rates were significantly higher than those of the non-progression group (2.2 and 0.6 per 100 Person-Years, respectively, *P*<0.001) (Table 3).

**Table 3:** All-cause and cardiovascular death rates according to the presence of aortic arch calcification (AoAC) at baseline and progression of AoAC.

	No. of events	Follow-up,	Event rate per
	/No. of patients	No. of Person-Years	100 Person-Years
**All-cause death**			
Baseline AoAC present group (*n* = 140)			
Progression (+)	27/90	136.3	19.8
Progression (−)	9/50	104.6	8.6
Baseline AoAC absent group (*n* = 223)			
Progression (+)	5/12	45.0	11.1
Progression (−)	19/211	863.3	2.2
**Cardiovascular death**			
Baseline AoAC present group (*n* = 140)			
Progression (+)	15/90	136.3	11.0
Progression (−)	4/50	105.2	3.8
Baseline AoAC absent group (*n* = 223)			
Progression (+)	2/12	45.4	4.4
Progression (−)	6/211	998.3	0.6

**Figure 2:**
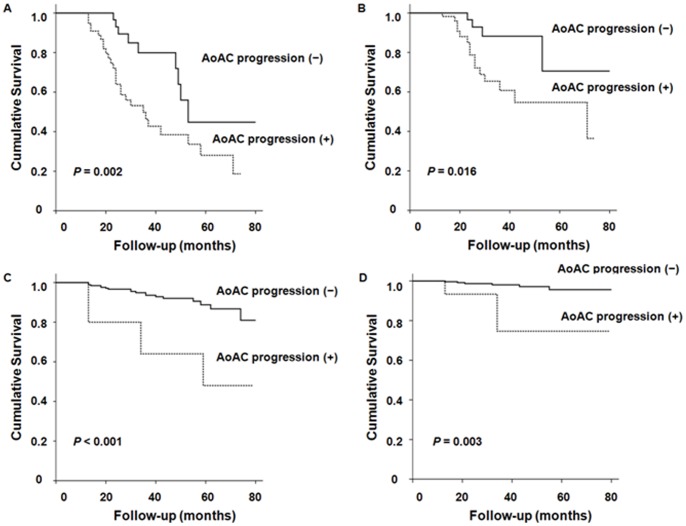
Kaplan-Meier analysis of aortic arch calcification (AoAC) progression for all-cause and cardiovascular mortality according to baseline AoAC subgroups. In baseline AoAC present group, patients with AoAC progression showed significantly higher all-cause (A) and cardiovascular (B) mortality (log-rank test, *P* = 0.002 and *P* = 0.016, respectively). Patients with AoAC progression in baseline AoAC absent group also showed significantly higher all-cause (C) and cardiovascular (D) mortality (*P*<0.001 and *P* = 0.003, respectively).

Kaplan-Meier analysis and Cox proportional hazard models (Figure 2 and Table 4) were used to determine the prognostic value of AoAC progression on mortality. In patients with baseline AoAC, patients with AoAC progression had significantly lower all-cause and cardiovascular mortality-free survival rates compared to patients without progression (log-rank test, *P* = 0.002 and 0.016, respectively). In addition, AoAC progression along with previous history of cardiovascular disease and hs-CRP levels was found to be significantly associated with all-cause and cardiovascular mortality in univariate Cox analysis. However, multivariate Cox proportional hazard analysis revealed that AoAC progression was an independent predictor of all-cause (HR: 2.625, 95% CI: 1.15–5.991, *P* = 0.022) and cardiovascular mortality (HR: 4.008, 95% CI: 1.079–14.890, *P* = 0.038). Similarly, in the subgroup of patients without baseline AoAC, Kaplan-Meier analysis showed that patients with AoAC progression had significantly higher risks for all-cause (*P*<0.001) and cardiovascular mortality (*P* = 0.003). Moreover, in univariate analysis, age, previous history of cardiovascular disease, the use of lipid-lowering drugs, and hs-CRP concentrations as well as AoAC progression were demonstrated to be significant risk factors for all-cause and cardiovascular mortality. However, subsequent multivariate Cox proportional hazard models found that AoAC progression was a significant independent predictor of all-cause mortality (HR: 3.408, 95% CI: 1.028–11.300, *P* = 0.045), but not of cardiovascular mortality (HR: 5.935, 95% CI: 0.912–36.995, *P* = 0.057).

**Table 4:** Cox’s proportional hazard models of aortic arch calcification (AoAC) progression for all-cause and cardiovascular mortality.

	Unadjusted		Adjusted	
	HR (95% CI)	*P*	HR (95% CI)	*P*
**Baseline AoAC present group (** ***n*** ** = 140)**				
All-cause mortality				
AoAC progression	2.679 (1.255–5.717)	0.011	a2.625 (1.15–5.991)	0.022
Cardiovascular mortality				
AoAC progression	3.506 (1.16–10.598)	0.026	a4.008 (1.079–14.890)	0.038
**Baseline AoAC absent group (** ***n*** ** = 223)**				
All-cause mortality				
AoAC progression	5.017 (1.853–13.587)	0.002	b3.408 (1.028–11.300)	0.045
Cardiovascular mortality				
AoAC progression	7.026 (1.408–35.053)	0.017	b5.935 (0.912–36.995)	NS

a Adjusted: adjusted for age, sex, presence of diabetes mellitus, previous cardiovascular disease, log high sensitivity C-reative protein, and albumin levels.

b Adjusted: adjusted for age, sex, presence of diabetes mellitus, previous cardiovascular disease, lipid-lowering therapy, log high sensitivity C-reactive protein, and albumin levels.

HR, hazard ratio; CI, confidence interval; NS, not significant.

## Discussion

Vascular calcification is common in ESRD patients and closely linked with cardiovascular disease, the leading cause of death in this population [1], [5], [8], [9], [10], [11]. In this study, we demonstrate that AoAC presence at the initiation of dialysis is a significant predictor for all-cause and cardiovascular mortality in a relatively large number of incident PD patients. In addition, AoAC progression was found to be associated with patient outcome, irrespective of the presence of AoAC at baseline.

Accumulating evidence has shown that vascular calcification is highly prevalent in ESRD patients [6], [7] and that it is associated with increased vascular stiffness and decreased vascular compliance, resulting in left ventricular (LV) hypertrophy and LV diastolic dysfunction [21], [22]. Furthermore, arterial stiffness leads to a decrease in diastolic blood pressure, which can compromise coronary perfusion to increase LV mass, irrespective of preexisting coronary artery disease [23], [24]. Based on these findings, some investigators have suggested that vascular calcification may contribute in part to significantly high cardiovascular mortality in ESRD. In accordance with most previous studies, this study showed AoAC presence at the start of PD was a significant independent predictor of all-cause and cardiovascular mortality in incident PD patients [3], [11], [18].

The prevalence of AoAC at baseline was 40.7% in this study, which was much lower than that of most previous studies from Western countries [2], [3], [13], [14], [25]. In the study by Ogawa et al [11], however, only 50.6% of 401 prevalent HD patients with dialysis duration of more than 8 years had AoAC. A study on 184 Korean incident dialysis patients also showed that AoAC was present in 41.3% before initial dialysis, which is comparable with the results of our study [26]. Taken together, the prevalence of vascular calcification in ESRD patients seems to be highly variable depending on not only the screening technique but also the studied population, such as ethnicity and BMI. Meanwhile, the proportion of smokers was significantly lower in patients with AoAC at baseline in this study. Most previous studies demonstrated that smoking was a significant risk factor for AoAC and that a dose-response relationship was observed between the amount of smoking and AoAC [27], [28]. Moreover, several studies revealed that smoking cessation decreased the risk of AoAC in some light ex-smokers [28], [29]. Considering these findings, we surmised that that the amount of smoking might be a possible explanation for the discrepancy in the association between smoking status and the risk of AoAC, and therefore re-evaluated the data of cigarette consumption and calculated pack-years of smoking in smokers. In result, compared to the baseline AoAC absent group, the mean amount of smoking was significantly higher in patients with baseline AoAC despite lower proportion of smokers. Furthermore, when smokers were dichotomized by the median value of the amount of smoking, the proportion of patients with AoAC at baseline was significantly higher in heavy smokers compared to light smoker group (26.2% vs. 13.4%, *P* = 0.04). Based on these findings, it was presumed that not only the smoking status but also the amount of smoking could affect the risk of AoAC. However, due to limited information about detailed smoking status (ex- or current smoker), the relationship of the smoking status and the amount of smoking with AoAC could not be thoroughly clarified in this study.

Compared to previous studies on the association of various parameters with vascular calcification and the clinical consequences of vascular calcification, the risk factors for the progression of vascular calcification are largely unexplored in dialysis patients. In addition, impacts of the vascular calcification progression on these patients’ outcome have not been elucidated. A previous study by Sigrist et al [14] investigated the independent factors associated with the progression of vascular calcification and the influence of it on mortality over 24 months in 134 patients with stage 4 and 5 CKD. It found that progressive calcification was associated with age, male gender, and serum alkaline phosphatase levels. Similarly, the NECOSAD study showed that age, hypercalcemia, hyperparathyroidism, and the interval between the first and last assessed AoACS were significantly linked with an increase in calcification score over time [3]. Kim et al [26] also found that age, dialysis duration, and the presence of AoAC were related to AoAC progression. However, in those studies, about two-thirds of patients were HD patients. In addition, changes in calcification score were significantly higher in HD patients than in PD patients. Moreover, the interval between the first and last measurement of AoACS was inconsistent in the NECOSAD study [3]. In this study, only incident PD patients were included and the interval between the first and follow-up AoACS assessment was 12 months in all patients. Therefore, the results of the aforementioned studies may not be applicable to ours. Even though age was significantly associated with AoAC progression in our subjects, when analysis was preformed separately according to the baseline AoAC presence, the association of age and AoACS with progression remained meaningful only in patients without baseline AoAC. Considering that age was significantly higher in patients with baseline AoAC than in patients without, we surmised that the effect of age on AoAC progression might be lessened in elderly incident PD patients who already had AoAC.

In the present study, AoAC progression was an independent predictor of unfavorable outcome in incident PD patients, which is in agreement with the results of most previous studies [3], [11], [18]. However, the mechanism by which AoAC progression influences mortality in ESRD patients has not been fully understood. We suppose that a different type of vascular calcification can be one of the possible mechanisms. London et al [9] examined the impact of intimal and medial calcification on the prognosis in prevalent HD patients and found that arterial medial calcification (AMC) was a much stronger predictor of mortality than arterial intimal calcification in these patients. On the other hand, it is well known that chronic inflammation, malnutrition, and atherosclerosis are closely linked with each other in ESRD patients [30]. Furthermore, the current study demonstrated that AoAC progression was observed even in patients without baseline AoAC and that AoAC progression was significantly associated with elevated hs-CRP levels in both baseline AoAC present and absent groups. Based on these findings, we surmised that AoAC progression was associated with AMC progression specific to dialysis therapy. A previous study also revealed that the reason of higher mortality in patients with AMC was attributed to increased arterial stiffness [31]. Increased arterial stiffness may cause vessel wall damage, atherosclerosis, and high pulse pressure, which were independent prognostic factors in ESRD [9], [31].

Mounting evidence has shown a close interrelationship among malnutrition, inflammation, and atherosclerosis [30]. In addition, atherosclerosis is closely associated with vascular and cardiac valvular calcification [32], [33]. Based on these findings, chronic inflammation has also been suggested to be implicated in the pathogenesis and progression of vascular calcification in dialysis patients and we also found that hs-CRP concentrations were significantly associated with the changes in AoACS in incident PD patients, irrespective of the presence of baseline AoAC. However, the association of hs-CRP levels with the progression of vascular calcification was not consistent. Previous studies demonstrated that CRP was independently associated with the progression of coronary artery calcification over a 24-month period in 40 prevalent HD patients and was identified as an independent risk factor for the progression of abdominal aortic calcification over 3 years in 71 prevalent HD patients [34], [35]. In contrast, other studies failed to identify association between CRP levels and the progression of vascular calcification in HD and/or PD patients [3], [36]. We surmised that failure to find this association was due to a small number of patients, combined analysis of patients with diverse dialysis modalities, and missing values. Since other circulating markers of inflammation and various calcification activators and inhibitors (such as bone morphogenetic proteins, matrix GIa-protein, fetuin-A, and osteoprotegerin) were not measured in this study [32], [37], [38], [39], our results that hs-CRP is the only non-traditional predictor of AoAC progression should be interpreted with caution.

### Conclusions

The present study shows that the presence of AoAC assessed by chest X-ray at the start of dialysis and the progression of AoAC during the first 12 months of dialysis were significant independent risk factors for mortality in incident PD patients. Taken together, regular follow-up by chest X-ray could be a simple and useful tool to stratify mortality risk in these patients. In addition, efforts to prevent development of vascular calcification and to attenuate progression of vascular calcification are needed to improve these patients’ outcomes.
